# Lignicolous freshwater fungi in Yunnan Province, China: an overview

**DOI:** 10.1080/21501203.2022.2058638

**Published:** 2022-04-03

**Authors:** Hong-Wei Shen, Dan-Feng Bao, Darbhe J. Bhat, Hong-Yan Su, Zong-Long Luo

**Affiliations:** a College of Agriculture and Biological Sciences, Dali University,Dali, Yunnan province, China; b Center of Excellence in Fungal Research, Mae Fah Luang University, Chiang Rai, Thailand; c School of Science, Mae Fah Luang University, Chiang Rai, Thailand; d Department of Entomology and Plant Pathology, Faculty of Agriculture, Chiang Mai University, Chiang Mai, Thailand

**Keywords:** Ascomycota, checklist, diversity, ecosystem functioning, lignicolous freshwater fungi

## Abstract

Yunnan Province is one of the rich biodiversity hotspots with abundant resources of lignicolous freshwater fungi. A total of 281 species of lignicolous freshwater fungi from 1986 to the present in Yunnan Province. They are mostly distributed in the classes *Dothideomycetes* and *Sordariomycetes*, a few species in the *Eurotiomycetes* and *Leotiomycetes*, and rarely reported in *Orbiliomycetes* and *Pezizomycetes*. Lignicolous freshwater fungi can decompose lignocellulose substrates and release energy and nutrients, and thus playing an important role in freshwater environment. This study briefly reviewed the biodiversity and taxonomic status of lignicolous freshwater fungi in Yunnan, the ecological functions of lignicolous freshwater fungi, factors affecting community distribution, application status, and research difficulties.

## Introduction

Freshwater fungi are a special ecological group that refers to fungi that whole or part of their life cycle is relied on freshwater habitats, including species that function in water and terrestrial fungi that release spores in water. (Wong et al. 1998; Luo et al. 2004a; Vijaykrishna et al. 2006). Various submerged substrates or sediments in freshwater, such as leaves, roots, wood, silt, mud, and other organic matter, serve as substrates for freshwater fungi (Luo et al. 2004a). Leaf-inhabiting freshwater fungi (the Ingoldian fungi) and lignicolous freshwater fungi (the wood decomposing fungi) have recently been increasingly studied in tropical and subtropical regions (Graça et al. 2016; Hyde et al. 2016a; Luo et al. 2019; Dong et al. 2020a).

Lignicolous freshwater fungi grow on submerged woody debris in freshwater environments such as streams, ponds, lakes, swamps, dams, and tree hollows (Wong et al. 1998; Luo et al. 2004a, 2019; Pinnoi et al. 2006; Pinruan et al. 2007, 2014; Hu et al. 2010b; Hyde et al. 2016a; Dong et al. 2020a). They represent a highly diverse taxonomic group, in which most of them are distributed in the classes *Dothideomycetes* and *Sordariomycetes* (Hyde et al. 2013; Wijayawardene et al. 2014; Maharachchikumbura et al. 2015; Luo et al. 2019; Dong et al. 2020a), a few species in *Eurotiomycetes* (Liu et al. 2015; Tian et al. 2016; Dong et al. 2018, 2020b; Wang et al. 2019) and *Orbiliomycetes* (Swe et al. 2009). These studies have mainly concentrated in tropical, subtropical and temperate regions. There have been many studies on lignicolous freshwater fungal communities (Tsui et al. 2000; Ho et al. 2001; Cai et al. 2002; Luo et al. 2004; Cai et al. 2006; Dhanasekaran et al. 2006; Hyde et al. 2016a). The diversity of lignicolous freshwater fungi in tropical regions is significantly higher than that in temperate regions (Graça et al. 2016; Hyde et al. 2016a). This may be due to the increased input of woody materials in streams from the neighbouring forests, and the increased diversity of plant substrates (Hyde et al. 2016a).

## Habitats of lignicolous freshwater fungi

The freshwater environment provides a unique habitat for lignicolous freshwater fungi. Wong et al. (1998) and Luo et al. (2004a) divided freshwater environments into three types: 1) Lentic (lakes, ponds, swamps, and pools), any natural aquatic environment lacking continuous flow, static, low or slow movement; 2) Lotic (rivers, streams, creeks and brooks), any natural aquatic environment with continuous flow of water; 3) other habitats including artificial water bodies (cooling tower, tree holes, etc.).

## Role and importance of lignicolous freshwater fungi in freshwater environments

Lignicolous freshwater fungi play an important role in the material and energy cycle of freshwater ecosystems. They can degrade the cellulose and lignocellulose of woody debris in the freshwater to form a soft rot (Koske and Duncan 1973; Zare-Maivan and Shearer 1988; Duarte et al. 2013). The degree of wood decay can be expressed in terms of weight loss, the number of soft rot cavities, and the reduction in compressive strength (Yuen et al. 1998). Eaton (1976) and Leightley and Eaton (1977) studied fungi on submerged wood in streams and cooling towers that can also degrade wood and found that produce soft rot holes in the wood test block in the laboratory. Although the role and importance of lignicolous freshwater fungi are unclear, it is an indisputable fact that they can decompose the extremely indigestible lignocellulose in submerged wood and release nutrients into the water (Yuen et al. 2000; Abdel-Raheem and Shearer 2002; Bucher et al. 2004).

## Diversity of lignicolous freshwater fungi in Yunnan Province

### Introduction of Yunnan Province

Yunnan is an inland province at a low latitude and high elevation, lying between 21°09’–29°15’ N and 97°32’–106°12’ E in southwestern China. The special geographical location has endowed Yunnan with abundant natural resources, such as the plateau cold-resistant biome in the west, and the tropical biome in the south and southwest. Yunnan is rich in freshwater resources, there are six major rivers, including four international rivers, *viz*. Dulongjiang River (Irrawaddy River), Nujiang River (Salween), Lancangjiang River (Mekong River) and Yuanjiang River (Red River), as well as two rivers in the territory, Nanpanjiang River (Pearl River) and Jinshajiang River (Yangtze River). Before flowing into the Pacific and Indian Oceans, tributaries of these six major rivers fan out to the south, the east and the southwest, forming a “broom-like shape”. The direction and location of the river are conducive to the entry of various species along the river valley into the watershed, making Yunnan with the most complicated watershed biological community in China. The abundant plateau lakes are also a major feature of Yunnan. There are 37 lakes over 1 square kilometre, with a total area of 1,164 square kilometres, a drainage area of 9,000 square kilometres, and a total storage capacity of about 29 billion cubic metres. These lakes are distributed in high-altitude areas, and most of them are depression pools formed by the subsidence of faults, with no water channels connected. There are moraine lakes and glacial eroded lakes in the alpine regions, with good water quality and abundant aquatic organisms (Yang et al. 2004). Because of their unique development and formation, and almost lakes are relatively isolated, each has its unique species (Yang et al. 1998, 2004). The special geographical location and complex geographical environment have formed a variety of ecosystems and habitats, breeding rich freshwater fungal resources.

### Diversity of lignicolous freshwater ascomycetes

Yang et al. (1985) investigated the diversity of freshwater fungi from Dianchi Lake in central Yunnan, and later, freshwater fungi were reported from Erhai and Changhu Lakes (Yang and Ding 1986; Yang et al. 1992). These researches focused on fungi that grow on sediments, water bodies and floating objects (litter, fallen leaves, flowers, fruits, protozoa, algae, dead aquatic animals and plants). These studies not only report a large number of freshwater fungi (many species are new records for China) but also discussed the impact of different water layers and seasons (dry and rainy seasons) on the distribution of freshwater fungi (Yang et al. 1986). It was not until 2000 that further research reports on lignicolous freshwater fungi appeared. Cai et al. (2002) systematically studied the lignicolous freshwater fungi in Fuxian Lake, reported 65 known species, and described a new species, *Pseudohalonectria fuxianii*. Among them, *Aniptodera chesapeakensis, Dictyosporium heptasporum, Massarina thalassioidea, Phaeoisaria clematidis, Pseudohalonectria lignicola* and *Savoryella lignicola* are dominant species. This study triggered a series of similar studies on lignicolous freshwater fungi in Yunnan Province. Luo et al. (2004a) studied freshwater fungi on submerged wood and grasses in Dianchi Lake, with 97 lignicolous freshwater fungi identified, and also discussed the influence of seasons, water pollution and hosts on the diversity of lignicolous freshwater fungi. Later, several studies reported lignicolous freshwater fungi in lotic environment (streams and rivers) of Yunnan (Cai and Hyde 2007c; Hu et al. 2010b; Liu et al. 2015; Su et al. 2015).

Since 2015, Yunnan has become one of the hotspots in lignicolous freshwater fungal research. The diversity of lignicolous freshwater fungi in streams and rivers in northwestern Yunnan has been well studied, resulting in the discovery of a large number of new species, new records and some highly diverse genera (e.g. *Acrogenospora, Dictyosporium, Distoseptispora, Pleurotheciella, Sporidesmium* and *Sporoschisma*) (Su et al. 2016a; Wang et al. 2016; Li et al. 2017; Luo et al. 2018a and 2018b;Zhao et al. 2018; Bao et al. 2020; Li et al. 2020a; Wan et al. 2021). Compared with the lotic freshwater environment, there are fewer studies on lignicolous freshwater fungi in lentic freshwater systems. Luo et al. (2004b) reviewed the freshwater fungi in mainland China and listed 68 lignicolous freshwater fungi in Yunnan. Hu et al. (2013) summarized the known data and comprehensively classified aquatic fungi of mainland China and listed 111 lignicolous freshwater fungi in Yunnan. *Sordariomycetes* and *Dothideomycetes* are the two largest classes of lignicolous freshwater ascomycetes that have been comprehensively reviewed (Luo et al. 2019; Dong et al. 2020a). This study listed 281 species of lignicolous freshwater fungi in Yunnan, which distributed in six classes, *viz. Dothideomycetes, Eurotiomycetes, Leotiomycetes, Orbiliomycetes, Pezizomycetes*, and *Sordariomycetes* (Table 1), the species affiliation in Table 1 is classified according to Hongsanan et al. (Hongsanan et al. 2020a and 2020b), Hyde et al. (2020c) and Wijayawardene et al. 2020. Amongst them, *Sordariomycetes* contains 134 species and of which *Chaetosphaeriaceae* is the richest family. *Dothideomycetes* contains 124 species. Fewer species have been reported in *Eurotiomycetes* (12 species) and *Leotiomycetes* (6 species). *Orbiliomycetes* and *Pezizomycetes* are rarely found in freshwater environments, one species in each class, *viz. Arthrobotrys dianchiensis* and *Aquapeziza globispora*, three species in *Ascomycota incertae sedis*.

**Table 1.** Checklist of lignicolous freshwater fungi in Yunnan Province, China

Species	References
***Dothideomycetes***	
***Pleosporomycetidae***	
***Pleosporales***	
***Amniculicolaceae***	
*Amniculicola aquatica* Z.L. Luo, K.D. Hyde & H.Y. Su	Hyde et al. 2019
*Amniculicola guttulata* Z.L. Luo, K.D. Hyde & H.Y. Su	Hyde et al. 2019
*Murispora aquatica* D.F. Bao, Z.L. Luo, K.D. Hyde & H.Y. Su	Bao et al. 2019b
*Murispora cicognanii* Wanas., Camporesi, E.B.G. Jones & K.D. Hyde	Hyde et al. 2019
*Murispora fagicola* Wanas., Camporesi, E.B.G. Jones & K.D. Hyde	Bao et al. 2019b
***Astrosphaeriellaceae***	
*Astrosphaeriella stellata* (Pat.) Sacc.	Luo et al. 2004a
*Pithomyces flavus* Berk. & Broome	Cai et al. 2002
*Xenoastrosphaeriella tornata* (Cooke) Jayasiri & K.D. Hyde	Cai et al. 2002
***Bambusicolaceae***	
*Bambusicola aquatica* W. Dong, H. Zhang & K.D. Hyde	Dong et al. 2020a
***Caryosporaceae***	
*Caryospora aquatica* H. Zhang, K.D. Hyde & Ariyaw	Dong et al. 2020
*Caryospora minima* Jeffers	Luo et al. 2004a
***Corynesporascaceae***	
*Corynespora lignicola* Z.L. Luo, H.Y. Su & K.D. Hyde	Hyde et al. 2020a
*Corynespora submersa* Z.L. Luo, H.Y. Su & K.D. Hyde	Hyde et al. 2020a
***Dictyosporiaceae***	
*Cheirosporium triseriale* L. Cai & K.D. Hyde	Cai et al. 2008
*Dictyocheirospora aquatica* Z.L. Luo, Bhat & K.D. Hyde	Wang et al. 2016
*Dictyocheirospora heptaspora* (Garov.) M.J. D'souza, Boonmee & K.D. Hyde	Wang et al. 2016
*Dictyocheirospora garethjonesii* Z.L. Luo, H.Y. Su & K.D. Hyde	Cai et al. 2002
*Dictyocheirospora rotunda* M.J. D'souza, Bhat & K.D. Hyde	Wang et al. 2016
*Dictyocheirospora tetraploides* (L. Cai & K.D. Hyde) J. Yang & K.D. Hyde	Cai et al. 2003a
*Dictyosporium biseriale* D.M. Hu, L. Cai & K.D. Hyde	Hu et al. 2010a
*Dictyosporium canisporum* L. Cai & K.D. Hyde	Cai et al. 2003a
*Dictyosporium lakefuxianense* L. Cai, K.D. Hyde & McKenzie	Cai et al. 2003b
*Dictyosporium polystichum* (Höhn.) Damon	Luo et al. 2004a
*Dictyosporium tetrasporum* L. Cai & K.D. Hyde	Cai and Hyde 2007a
*Dictyosporium yunnanense* L. Cai, K.D. Hyde & McKenzie	Cai et al. 2003b
*Digitodesmium heptasporum* L. Cai & K.D. Hyde	Cai et al. 2003a
*Aquadictyospora lignicola* Z.L. Luo, W.L. Li, K.D. Hyde & H.Y. Su	Li et al. 2017
*Jalapriya pulchra* M.J. D'souza, H.Y. Su, Z.L. Luo & K.D. Hyde	Boonmee et al. 2012
*Pseudodictyosporium wauense* Matsush	Li et al. 2021
*Vikalpa lignicola* M.J. D'souza, Bhat, H.Y. Su & K.D. Hyde	Boonmee et al. 2016
***Lentitheciaceae***	
*Lentithecium cangshanense* Z.L. Luo, X.J. Su & K.D. Hyde	Su et al. 2016b
*Lentithecium kunmingense* W. Dong, H. Zhang & K.D. Hyde	Dong et al. 2020a
*Setoseptoria arundinacea* (Sowerby) Kaz. Tanaka & K. Hiray.	Luo et al. 2004a
***Lindgomycetaceae***	
*Aquimassariosphaeria kunmingensis* W. Dong, Doilom & K.D. Hyde	Dong et al. 2020a
*Clohesyomyces aquaticus* K.D. Hyde	Dong et al. 2020a
***Lophiostomataceae***	
*Biappendiculispora japonica* Thambug., Wanas., Kaz. Tanaka & K.D. Hyde	Bao et al. 2019a
*Flabellascoma aquaticum* D.F. Bao, Z.L. Luo, K.D. Hyde & H.Y. Su	Bao et al. 2019a
*Flabellascoma fusiforme* D.F. Bao, Z.L. Luo, K.D. Hyde & H.Y. Su	Bao et al. 2019a
*Lentistoma bipolare* (K.D. Hyde) A. Hashim., K. Hiray. & Kaz. Tanaka	Luo et al. 2004a
*Neovaginatispora fuckelii* (Sacc.) A. Hashim., K. Hiray. & Kaz. Tanaka	Bao et al. 2019a
*Pseudocapulatispora longiappendiculata* Mapook & K.D. Hyde	Dong et al. 2020a
*Sigarispora clavata* D.F. Bao, Z.L. Luo, K.D. Hyde & H.Y. Su	Bao et al. 2019a
***Massarinaceae***	
*Helminthosporium aquaticum* H.Y. Su, Z.L. Luo & K.D. Hyde	Zhu et al. 2016
*Helminthosporium velutinum* Link	Zhu et al. 2016
***Melanommataceae***	
*Camposporium appendiculatum* D.F. Bao, Z.L. Luo, K.D. Hyde & H.Y. Su	Hyde et al. 2020b
*Camposporium multiseptatum* D.F. Bao, Z.L. Luo, K.D. Hyde & H.Y. Su	Hyde et al. 2020b
*Camposporium pellucidum* (Grove) S. Hughes	Hyde et al. 2020b
*Phragmocephala atra* (Berk. & Broome) E.W. Mason & S. Hughes	Su et al. 2015
*Phragmocephala garethjonesii* H.Y. Su, Udayanga & K.D. Hyde	Su et al. 2015
***Morosphaeriaceae***	
*Aquihelicascus thalassioideus* (K.D. Hyde & Aptroot) W. Dong & H. Zhang	Luo et al. 2004a
*Aquihelicascus yunnanensis* W. Dong, H. Zhang & K.D. Hyde	Dong et al. 2020a
*Neohelicascus aquaticus* (H. Zhang & K.D. Hyde) W. Dong, K.D. Hyde & H. Zhang	Dong et al. 2020a
*Neohelicascuselaterascus* (Shearer) W. Dong, K.D. Hyde & H. Zhang	Luo et al. 2004a
*Neohelicascus submersus* H. Yang, W. Dong, K.D. Hyde & H. Zhang	Dong et al. 2020a
***Nigrogranaceae***	
*Nigrograna cangshanensis* Z.L. Luo, H.Y. Su & K.D. Hyde	Tibpromma et al. 2017
***Occultibambusaceae***	
*Occultibambusa kunmingensis* C.X. Liu, H. Zhang & K.D. Hyde	Dong et al. 2020a
*Occultibambusa pustula* D.Q. Dai & K.D. Hyde	Dong et al. 2020a
*Seriascoma didymosporum* Phook., D.Q. Dai, Karun. & K.D. Hyde	Dong et al. 2020a
***Periconiaceae***	
*Periconia aquatica* Z.L. Luo, H.Y. Su & K.D. Hyde	Hyde et al. 2017
*Periconia byssoides* Pers.	Luo et al. 2004a
*Periconia digitata* (Cooke) Sacc	Luo et al. 2004a
*Periconia minutissima* Corda	Luo et al. 2004a
*Periconia submersa* Z.L. Luo, H.Y. Su & K.D. Hyde	Hyde et al. 2017
***Phaeoseptaceae***	
*Pleopunctum pseudoellipsoideum* N.G. Liu, K.D. Hyde & J.K. Liu	Dong et al. 2020a
***Pleomassariaceae***	
*Beverwykella pulmonaria* (Beverw.) Tubaki	Cai et al. 2002
***Pleosporaceae***	
*Curvularia eragrostidis* (Henn.) J.A. Mey	Su et al. 2015
*Curvularia verruculosa* Tandon & Bilgrami ex M.B. Ellis	Su et al. 2015
***Pseudoastrosphaeriellaceae***	
*Pseudoastrosphaeriella papillata* (K.D. Hyde & J. Fröhl.) Phook. & K.D. Hyde	Luo et al. 2004a
***Pseudoberkleasmiaceae***	
*Pseudoberkleasmium chiangmaiense* Y.Z. Lu & K.D. Hyde	Bao et al. 2021
***Roussoellaceae***	
*Neoroussoella bambusae* Phook., Jian K. Liu & K.D. Hyde	Dong et al. 2020a
*Neoroussoella leucaenae* Jayasiri, E.B.G. Jones & K.D. Hyde	Dong et al. 2020a
*Roussoella aquatica* W. Dong, H. Zhang & K.D. Hyde	Dong et al. 2020a
***Tetraplosphaeriaceae***	
*Shrungabeeja vadirajensis* V.G. Rao & K.A. Reddy	Zhang et al. 2019
*Tetraploa aquatica* W.L. Li & H.Y. Su	Li et al. 2020a
*Tetraploa puzheheiensis* W. Dong, H. Yang & H. Zhang	Dong et al. 2020a
*Tetraploa yunnanensis* W. Dong, H. Yang & H. Zhang	Dong et al. 2020a
***Torulaceae***	
*Dendryphion aquaticum* H. Y. Su & K.D. Hyde	Su et al. 2016a
*Dendryphion fluminicola* Z.L. Luo, D.J. Bhat & K.D. Hyde	Su et al. 2018
*Dendryphion hydei* J.F. Li, Phookamsak & Jeewon	Boonmee et al. 2021
*Dendryphion nanum* (Nees) S. Hughes	Su et al. 2016a
*Dendryphion submersum* H.Y. Su & K.D. Hyde	Su et al. 2016a
*Neotorula aquatica* Z.L. Luo & K.D. Hyde	Su et al. 2016a
*Neotorula submersa* Z.L. Luo, H.Y. Su & K.D. Hyde	Su et al. 2016a
*Rostriconidium aquaticum* Z.L. Luo, K.D. Hyde & H.Y. Su	Su et al. 2018
*Rostriconidium cangshanensis* H.W. Shen, Z.L. Luo & H.Y. Su	Shen et al. 2021a
*Rostriconidium pandanicola* Tibpromma & K.D. Hyde	Shen et al. 2021a
*Torula aquatica* Z.L. Luo, K.D. Hyde, X.J. Su & H.Y. Su	Su et al. 2018
*Torula fici* Crous	Su et al. 2018
*Torula lancangjiangensis* H.W. Shen, S. Boonmee, Z.L. Luo & K.D. Hyde	Boonmee et al. 2021
*Torula mackenziei* J.F. Li, Phook. & K.D. Hyde	Boonmee et al. 2021
*Torula masonii* Crous	Su et al. 2018
***Trematosphaeriaceae*** *Hadrospora fallax* (Mouton) Boise	Luo et al. 2004a
***Pleosporales*** genera *incertae sedis*	
*Ascorhombispora aquatica* L. Cai & K.D. Hyde	Cai and Hyde 2007b
***Dothideomycetes*** orders *incertae sedis*	
***Botryosphaeriales***	
***Botryosphaeriaceae***	
*Tiarosporella paludosa* (Sacc. & Fiori) Höhn	Luo et al. 2004a
***Jahnulales***	
***Aliquandostipitaceae***	
*Brachiosphaera tropicalis* Nawawi	Cai et al. 2002
*Jahnula granulosa* K.D. Hyde & S.W. Wong	Cai et al. 2002
*Jahnula poonythii* K.D. Hyde & S.W. Wong	Cai et al. 2002
*Jahnula rostrata* Raja & Shearer	Dong et al. 2020a
*Xylomyces chlamydosporus* Goos, R.D. Brooks & Lamore	Luo et al. 2004a
*Xylomyces pusillus* Goh, W.H. Ho, K.D. Hyde & C.K.M. Tsui	Cai et al. 2002
***Kirschsteiniotheliales***	
***Kirschsteiniotheliaceae***	
*Kirschsteiniothelia aethiops* (Sacc.) D. Hawks	Su et al. 2016a
*Kirschsteiniothelia aquatica* Z.L. Luo, K.D. Hyde & H.Y. Su	Bao et al. 2018
*Kirschsteiniothelia cangshanensis* Z.L. Luo, D.F. Bao, K.D. Hyde & H.Y. Su	Bao et al. 2018
*Kirschsteiniothelia fluminicola* Z.L. Luo, K.D. Hyde & H.Y. Su	Bao et al. 2018
*Kirschsteiniothelia rostrata* Jing Yang & K.D. Hyde	Bao et al. 2018
*Kirschsteiniothelia submersa* H.Y. Su & K.D. Hyde	Su et al. 2016a
***Minutisphaerales***	
***Acrogenosporaceae***	
*Acrogenospora aquatica* D.F. Bao, Z.L. Luo, K.D. Hyde & H.Y. Su	Bao et al. 2020
*Acrogenospora basalicellularispora* D.F. Bao, Z.L. Luo, K.D. Hyde & H.Y. Su	Bao et al. 2020
*Acrogenospora ellipsoidea* D.M. Hu, L. Cai & K.D. Hyde	Hu et al. 2010a
*Acrogenospora guttulatispora* D.F. Bao, Z.L. Luo, K.D. Hyde & H.Y. Su	Bao et al. 2020
*Acrogenospora obovoidspora* D.F. Bao, Z.L. Luo, K.D. Hyde & H.Y. Su	Bao et al. 2020
*Acrogenospora olivaceospora* D.F. Bao, Z.L. Luo, K.D. Hyde & H.Y. Su	Bao et al. 2020
*Acrogenospora submersa* D.F. Bao, Z.L. Luo, K.D. Hyde & H.Y. Su	Bao et al. 2020
*Acrogenospora subprolata* Goh, K.D. Hyde & C.K.M. Tsui	Bao et al. 2020
*Acrogenospora verrucispora* Hong Zhu, L. Cai & K.Q. Zhang	Bao et al. 2020
*Acrogenospora yunnanensis* D.F. Bao, Z.L. Luo, K.D. Hyde & H.Y. Su	Bao et al. 2020
***Tubeufiales***	
***Tubeufiaceae***	
*Muripulchra aquatica* Z.L. Luo, H.Y. Su & K.D. Hyde	Luo et al. 2017
*Neohelicomyces aquaticus* Z.L. Luo, Bhat & K.D. Hyde	Luo et al. 2017
*Neohelicomyces dehongensis* H. Zhang, W. Dong & K.D. Hyde	Dong et al. 2020a
*Neohelicomyces grandisporus* Z.L. Luo, Boonmee & K.D. Hyde	Luo et al. 2017
*Neohelicomyce submersus* Z.L. Luo, H.Y. Su & K.D. Hyde	Luo et al. 2017
*Pseudohelicomyces hyalosporus* Y.Z. Lu, J.K. Liu & K.D. Hyde	Luo et al. 2017
*Tubeufia aquatica* Z.L. Luo, Bhat & K.D. Hyde	Luo et al. 2017
*Tubeufia cylindrothecia* (Seaver) Höhn.	Luo et al. 2017
***Eurotiomycetes***	
***Chaetothyriales***	
***Herpotrichiellaceae***	
*Minimelanolocus clavatus* Y.L. Wan, D.F. Bao & H.Y. Su	Wan et al. 2021
*Minimelanolocus nujiangensis* Y.L. Wan, Z.L. Luo & H.Y. Su	Wan et al. 2021
*Minimelanolocus submerses* Z.L. Luo, H.Y. Su & K.D. Hyde	Hyde et al. 2016b
*Minimelanolocus yunnanensis* Q Tian & K.D. Hyde	Tian et al. 2016
*Thysanorea asiatica* (H.Y. Su, Udayanga & K.D. Hyde) Hern.-Restr. & Crous	Liu et al. 2015
*Thysanorea curvata* (H.Y. Su, Udayanga & K.D. Hyde) Hern.-Restr. & Crous	Liu et al. 2015
*Thysanorea melanica* (H.Y. Su, Udayanga & K.D. Hyde) Hern.-Restr. & Crous	Liu et al. 2015
*Thysanorea obscura* (Matsush.) Hern.-Restr. & Crous	Liu et al. 2015
*Thysanorea yunnanensis* Hern.-Restr. & Crous	Liu et al. 2015
***Chaetothyriales*** genera *incertae sedis*	
*Uncispora sinensis* G.Z. Yang & Z.F. Yu	Yang et al. 2011
*Uncispora wuzhishanensis* L.P. Chen & Z.F. Yu	Liu et al. 2018
***Sclerococcales***	
***Dactylosporaceae***	
*Pseudobactrodesmium aquaticum* W. Dong, H. Zhang & K.D. Hyde	Dong et al. 2020b
***Leotiomycetes***	
***Helotiales***	
***Discinellaceae***	
*Tetrachaetum elegans* Ingold	Yang and Ding 1986
***Helotiales*** genera *incertae sedis*	
*Dactylaria hoogi* R.F. Castañeda & W.B. Kendr.	Cai et al. 2002
*Dactylaria longidentata* Cazau, Aramb. & Cabello	Luo et al. 2004a
*Dactylaria splendida* R.F. Castañeda & W.B. Kendr.	Luo et al. 2004a
*Dactylaria triseptata* (Matsush.) R.F. Casta–eda & W.B. Kendr.	Luo et al. 2004a
*Dactylaria uniseptate* Matsush.	Cai et al. 2002
***Orbiliomycetes***	
***Orbiliales***	
***Orbiliaceae***	
*Arthrobotrys dianchiensis* (Y. Hao & K.Q. Zhang) Z.F. Yu	Hao et al. 2004
***Pezizomycetes***	
***Pezizales***	
***Pezizaceae***	
*Aquapeziza globispora* D.M. Hu, L. Cai & K.D. Hyde	Hu et al. 2012c
***Sordariomycetes***	
***Diaporthomycetidae***	
***Annulatascales***	
***Annulatascaceae***	
*Annulatascus fusiformis* K.D. Hyde & S.W. Wong	Cai et al. 2006
*Annulatascus menglensis* D.M. Hu, L. Cai & K.D. Hyde	Hu et al. 2012a
***Annulatascales*** genus *incertae sedis*	
*Clohiesia curvispora* L. Cai & K.D. Hyde	Cai et al. 2007c
***Atractosporales***	
***Atractosporaceae***	
*Atractospora aquatica* Z.L. Luo, K.D. Hyde & H.Y. Su	Luo et al. 2019
*Atractospora ellipsoidea* (W.H. Ho, C.K.M. Tsui, Hodgkiss & K.D. Hyde) Réblová & J. Fourn.	Dong et al. 2021b
***Conlariaceae***	
*Conlarium aquaticum* W. Dong, H. Zhang & K.D. Hyde	Dong et al. 2021b
***Distoseptisporales***	
***Aquapteridosporaceae***	
*Aquapteridospora fusiformis Z*.L. Luo, D.F. Bao, H.Y. Su & K.D. Hyde	Luo et al. 2019
***Distoseptisporaceae***	
*Distoseptispora aquatica* Z.L. Luo, H. Y. Su & K.D. Hyde	Su et al. 2016a
*Distoseptispora cangshanensis* Z.L. Luo, K.D. Hyde & H.Y. Su	Luo et al. 2018b
*Distoseptispora clematidis* Phukhams., M.V. de Bult & K.D. Hyde	Shen et al. 2021b
*Distoseptispora euseptata* W.L. Li, H.Y. Su & J.K. Liu	Li et al. 2021
*Distoseptispora fluminicola* McKenzie, HY. Su, Z.L. Luo & K.D. Hyde	Su et al. 2016a
*Distoseptispora lancangjiangensis* H.W Shen, H.Y. Su, K.D. Hyde & Z.L. Luo	Shen et al. 2021b
*Distoseptispora longispora* H.Y. Song & D.M. Hu	Song et al. 2020
*Distoseptispora obpyriformis* Z.L. Luo & H.Y. Su	Luo et al. 2018b
*Distoseptispora rostrata* Z.L. Luo, K.D. Hyde & H.Y. Su	Luo et al. 2018b
*Distoseptispora submersa* Z.L. Luo, K.D. Hyde & H.Y. Su	Luo et al. 2018b
*Distoseptispora suoluoensis* J. Yang, Maharachch. & K.D. Hyde	Luo et al. 2018b
*Distoseptispora thysanolaenae* Goonas., Dayarathne, Phookamsak & K.D.Hyde	Shen et al. 2021b
*Distoseptispora yunnanensis* W.L. Li, H.Y. Su & Jian K. Liu	Li et al. 2021
***Magnaporthales***	
***Ceratosphaeriaceae***	
*Ceratosphaeria aquatica* Z.L. Luo, K.D. Hyde & H.Y. Su	Luo et al. 2019
***Magnaporthaceae***	
*Aquafiliformis lignicola* Z.L. Luo, K.D. Hyde & H.Y. Su	Luo et al. 2019
***Ophioceraceae***	
*Ophioceras aquaticum* D.M. Hu, L. Cai & K.D. Hyde	Hu et al. 2012b
*Ophioceras guttulatum* C.K.M. Tsui, H.Y.M. Leung, K.D. Hyde & Hodgkiss	Cai et al. 2006
***Pseudohalonectriaceae***	
*Pseudohalonectria fuxianii* L. Cai, C.K.M. Tsui, K.Q. Zhang & K.D. Hyde	Cai et al. 2002
*Pseudohalonectria lignicola* Minoura & T. Muroi	Cai et al. 2002
*Pseudohalonectria lutea* Shearer	Cai et al. 2002
***Myrmecridiales***	
***Myrmecridiaceae***	
*Myrmecridium aquaticum* Z.L. Luo, K.D. Hyde & H.Y. Su	Luo et al. 2019
***Sporidesmiales***	
***Sporidesmiaceae***	
*Sporidesmium aturbinatum* (S. Hughes) M.B. Ellis	Bao et al. 2021
*Sporidesmium brachypus* (Ellis & Everh.) S. Hughes	Luo et al. 2019
*Sporidesmium cangshanense* Z.L. Luo & K.D. Hyde	Su et al. 2016a
*Sporidesmium dulongense* Z.L. Luo, K.D. Hyde & H.Y. Su	Hyde et al. 2020c
*Sporidesmium fluminicola* H. Y. Su & K.D. Hyde	Su et al. 2016a
*Sporidesmium lageniforme* Z.L. Luo, K.D. Hyde & H.Y. Su	Luo et al. 2019
*Sporidesmium lignicola* Z.L. Luo, K.D. Hyde & H.Y. Su	Luo et al. 2019
*Sporidesmium nujiangense* D.F. Bao, H.Y. Su, K.D. Hyde & Z.L. Luo	Bao et al. 2021
*Sporidesmium submersum* H.Y. Su & K.D. Hyde	Su et al. 2016a
*Sporidesmium tropicale* M.B. Ellis	Bao et al. 2021
***Togniniales***	
***Togniniaceae***	
*Phaeoacremonium aquaticum* (D.M. Hu, L. Cai & K.D. Hyde) Gramaje, L. Mostert & Crous	Hu et al. 2012b
*Phaeoacremonium ovale* S.K. Huang, R. Jeewon & K.D. Hyde	Huang et al. 2018
***Xenospadicoidales***	
***Xenospadicoidaceae***	
*Neospadicoides aquatica* Z.L. Luo, K.D. Hyde & H.Y. Su	Luo et al. 2019
*Neospadicoides lignicola* Z.L. Luo, K.D. Hyde & H.Y. Su	Luo et al. 2019
*Neospadicoides yunnanensis* Z.L. Luo, K.D. Hyde & H.Y. Su	Luo et al. 2019
*Spadicoides bambusicola* D.Q. Zhou, Goh & K.D. Hyde	Cai et al. 2006
*Spadicoides minuta* L. Cai, McKenzie & K.D. Hyde	Cai et al. 2004
***Diaporthomycetidae*** families *incertae sedis*	
***Barbatosphaeriaceae***	
*Barbatosphaeria lignicola* Z.L. Luo, H.Y. Su & K.D. Hyde	Luo et al. 2019
***Papulosaceae***	
*Wongia aquatica* Z.L. Luo, K.D. Hyde & H.Y. Su	Luo et al. 2019
*Wongia fusiformis* D.F. Bao, H.Y. Su, K.D. Hyde & Z.L. Luo	Bao et al. 2021
***Rhamphoriaceae***	
*Rhodoveronaea aquatica* Z.L. Luo, K.D. Hyde & H.Y. Su	Luo et al. 2019
***Diaporthomycetidae*** genera *incertae sedis*	
***Pseudostanjehughesia***	
*Pseudostanjehughesia lignicola* Z.L. Luo, K.D. Hyde & H.Y. Su	Luo et al. 2019
***Hypocreomycetidae***	
***Coronophorales***	
***Coronophorales*** genera *incertae sedis*	
*Papulaspora sepedonioides* Preuss	Cai et al. 2006
***Glomerellales***	
***Reticulascaceae***	
*Cylindrotrichum clavatum* W. Gams	Maharachchikumbura et al. 2018
*Cylindrotrichum gorii* Lunghini	Maharachchikumbura et al. 2018
*Cylindrotrichum submersum* Z.L. Luo, H.Y. Su & K.D. Hyde	Luo et al. 2019
*Kylindria aquatica* Z.L. Luo, Maharachch. & Cheew	Maharachchikumbura et al. 2018
*Kylindria chinensis* Maharachch., H.Y. Su & Cheew	Maharachchikumbura et al. 2018
***Hypocreales***	
***Nectriaceae***	
*Aquanectria jacinthicolor* S.K. Huang, R. Jeewon & K.D. Hyde	Huang et al. 2018
*Aquanectria penicillioides* (Ingold) L. Lombard & Crous	Luo et al. 2019
*Chaetopsina beijingensis* Crous & Y. Zhang ter	Luo et al. 2019
*Cosmospora aquatica* Z.L. Luo, H.Y. Su & K.D. Hyde	Luo et al. 2019
*Mariannaea cinerea* D.M. Hu & L. Cai	Hu et al. 2017
*Mariannaea samuelsii* Seifert & Bissett	Luo et al. 2019
*Mariannaea superimposita* (Matsush.) Samuels	Luo et al. 2019
*Paracremonium binnewijzendii* Houbraken, van der Kleij & L. Lombard	Luo et al. 2019
*Payosphaeria minuta* H.Y.M. Leung	Cai et al. 2006
***Niessliaceae***	
*Paraniesslia aquatica* L. Cai & K.D. Hyde	Cai et al. 2007c
***Stachybotryaceae***	
*Stachybotrys chartarum* (Ehrenb.) S. Hughes	Luo et al. 2019
*Stachybotrys chlorohalonatus* B. Andersen & Thrane	Luo et al. 2019
***Microascales***	
***Halosphaeriaceae***	
*Natantispora retorquens* (Shearer & J.L. Crane) J. Campb., J.L. Anderson & Shearer	Cai et al. 2002
*Phaeonectriella lignicola* R.A. Eaton & E.B.G. Jones	Luo et al. 2004a
***Pleosporomycetidae***	
***Pleosporales***	
***Melanommataceae***	
*Sporidesmiella aquatica* Z.L. Luo, K.D. Hyde & H.Y. Su	Luo et al. 2019
*Sporidesmiella hyalosperma* (Corda) P.M. Kirk	Dong et al. 2020a
*Sporidesmiella novae-zelandiae* (S. Hughes) Madrid, Hern.-Restr. & Crous	Luo et al. 2019
***Savoryellomycetidae***	
***Conioscyphales***	
***Conioscyphaceae***	
*Conioscypha aquatica* Z.L. Luo, K.D. Hyde & H.Y. Su	Luo et al. 2019
*Conioscypha submersa* Z.L. Luo, K.D. Hyde & H.Y. Su	Luo et al. 2019
***Pleurotheciales***	
***Pleurotheciaceae***	
*Phaeoisaria aquatica* Z.L. Luo, X.J. Su & K.D. Hyde	Luo et al. 2018a
*Phaeoisaria clematidis* (Fuckel) S. Hughes	Luo et al. 2018a
*Pleurotheciella aquatica* Z.L. Luo, D.J. Bhat, H.Y. Su & K.D. Hyde	Luo et al. 2018a
*Pleurotheciella fusiformis* Z.L. Luo, H.Y. Su & K.D. Hyde	Luo et al. 2018a
*Pleurotheciella guttulate* Z.L. Luo, H.Y. Su & K.D. Hyde	Luo et al. 2018a
*Pleurotheciella lunata* Z.L. Luo, D.J. Bhat & K.D. Hyde	Luo et al. 2018a
*Pleurotheciella saprophytica* Z.L. Luo, H.Y. Su & K.D. Hyde	Luo et al. 2018a
*Pleurotheciella submersa* Z.L. Luo & K.D. Hyde	Luo et al. 2018a
*Pleurotheciella uniseptate* (Matsush.) Seifert	Luo et al. 2018a
*Pleurothecium aquaticum* Z.L. Luo, H.Y. Su & K.D. Hyde	Luo et al. 2018a
*Pleurothecium pulneyense* Subram. & Bhat	Luo et al. 2018a
*Pleurothecium recurvatum* (Morgan) Höhn.	Luo et al. 2019
*Saprodesmium dematiosporum* W. Dong, Doilom & K.D. Hyde	Dong et al. 2021a
*Sterigmatobotrys uniseptatus* H.S. Chang	Luo et al. 2019
***Savoryellales***	
***Savoryellaceae***	
*Canalisporium jinghongense* L. Cai, K.D. Hyde & McKenzie	Cai et al. 2003b
*Dematiosporium aquaticum* Z.L. Luo, K.D. Hyde & H.Y. Su	Luo et al. 2019
***Sordariomycetidae***	
***Chaetosphaeriales***	
***Chaetosphaeriaceae***	
*Anacraspedodidymum submersum* Z.F. Yu & R.F.	Zheng et al. 2021
*Chaetosphaeria aquatica* Z.L. Luo, K.D. Hyde & H.Y. Su	Luo et al. 2019
*Chaetosphaeria catenulata* Z.L. Luo, K.D. Hyde & H.Y. Su	Luo et al. 2019
*Chaetosphaeria cubensis* Hol.-Jech.	Luo et al. 2019
*Chaetosphaeria guttulate* Z.L. Luo, K.D. Hyde & H.Y. Su	Luo et al. 2019
*Chaetosphaeria myriocarpa* (Fr.) C. Booth	Luo et al. 2019
*Chaetosphaeria submersa* Z.L. Luo, K.D. Hyde & H.Y. Su	Luo et al. 2019
*Chloridium gonytrichii* (F.A. Fernández &Huhndorf) Réblová & Seifert	Luo et al. 2019
*Codinaea yunnanensis* Z.L. Luo, K.D. Hyde & H.Y. Su	Luo et al. 2019
*Dictyochaeta cangshanensis* Z.L. Luo, K.D. Hyde & H.Y. Su	Luo et al. 2019
*Dictyochaeta ellipsoidea* Z.L. Luo, K.D. Hyde & H.Y. Su	Luo et al. 2019
*Dictyochaeta lignicola* Z.L. Luo, H.Y. Su & K.D. Hyde	Luo et al. 2019
*Dictyochaeta submersa* Z.L. Luo, K.D. Hyde & H.Y. Su	Luo et al. 2019
*Exserticlava yunnanensis* L. Cai & K.D. Hyde	Cai and Hyde 2007b
*Sporoschisma hemipsilum* (Berk. & Broome) Zelski, A.N. Mill. & Shearer	Luo et al. 2016
*Sporoschisma juvenile* Boud	Luo et al. 2019
*Sporoschisma mirabile* Berk. & Broome	Luo et al. 2016
*Sporoschism anigroseptatum* D. Rao & P.Rag. Rao	Luo et al. 2016
*Sporoschisma phaeocentron* W.H. Ho, K.D. Hyde & Goh	Luo et al. 2016
*Sporoschisma taitense* (Mugambi & Huhndorf) A.N. Mill.	Luo et al. 2016
*Tainosphaeria lunata* Z.L. Luo, K.D. Hyde & H.Y. Su	Luo et al. 2019
***Helminthosphaeriaceae***	
*Hilberina breviseta* (P. Karst.) Huhndorf & A.N. Mill.	Luo et al. 2004a
***Coniochaetales***	
***Cordanaceae***	
*Cordana aquatica* Z.L. Luo, K.D. Hyde & H.Y. Su	Luo et al. 2019
*Cordana lignicola* Z.L. Luo, K.D. Hyde & H.Y. Su	Luo et al. 2019
*Cordana terrestris* (Timonin) Hern.-Restr., Gené & Guarro	Luo et al. 2019
*Cordana uniseptata* L. Cai, McKenzie & K.D. Hyde	Cai et al. 2004
***Sordariales***	
***Chaetomiaceae***	
*Chaetomium globosum* Kunze	Luo et al. 2019
*Trichocladium lignicola* I. Schmidt	Cai et al. 2002
***Lasiosphaeriaceae***	
*Cercophora caudata* (Sacc.) N. Lundq.	Luo et al. 2019
*Zopfiella latipes* (N. Lundq.) Malloch & Cain	Cai et al. 2002
***Sordariales*** genera *incertae sedis*	
*Cuspidatispora xiphiago* Shearer & Bartolata	Luo et al. 2019
***Xylariomycetidae***	
***Amphisphaeriales***	
***Apiosporaceae***	
*Arthrinium aquaticum* Z.L. Luo, K.D. Hyde & H.Y. Su	Luo et al. 2019
***Sporocadaceae***	
*Hymenopleella lakefuxianensis* (L. Cai, Jeewon & K.D. Hyde) F. Liu, L. Cai & Crous	Jeewon et al. 2003
*Seiridium aquaticum* Z.L. Luo, K.D. Hyde & H.Y. Su	Luo et al. 2019
***Xylariales***	
***Hypoxylaceae***	
*Hypoxylon lignicola* Z.L. Luo, K.D. Hyde & H.Y. Su	Luo et al. 2019
***Xylariaceae***	
*Vamsapriya aquatica* D.F. Bao, H.Y. Su, K.D. Hyde & Z.L. Luo	Bao et al. 2021
*Vamsapriya indica* Gawas & Bhat	Bao et al. 2021
***Sordariomycetes*** families *incertae sedis*	
***Acrodictyaceae***	
*Acrodictys fluminicola* Z.L. Luo, K.D. Hyde & H.Y. Su	Luo et al. 2019
***Junewangiaceae***	
*Dictyosporella ellipsoidea* W. Dong, H. Zhang & K.D. Hyde	Dong et al. 2021b
*Dictyosporella hydei* H.Y. Song & D.M. Hu	Song et al. 2018a
*Junewangia aquatica* H.Y. Song & D.M. Hu	Song et al. 2018b
***Sordariomycetes*** genera *incertae sedis*	
*Ascoyunnania aquatica* L. Cai & K.D. Hyde	Cai et al. 2005
***Ascomycota****incertae sedis*	
*Candelabrum brocchiatum* Tubaki	Luo et al. 2004a
*Pseudofuscophialis lignicola* Sivan. & H.S. Chang	Cai and Hyde 2007b
*Vanakripa menglensis* D.M. Hu, L. Cai & K.D. Hyde	Hu et al. 2010

A comprehensive study on the diversity and community distribution pattern of lignicolous freshwater fungi in northwestern Yunnan has resulted in abundant species resources (Luo et al. 2019). In recent years, lignicolous freshwater fungi are being investigated in Yunnan, including six major water systems, namely, Dulongjiang River (Irrawaddy River), Nujiang River (Salween River), Lancnagjiang River (Mekong River), Yuanjiang River (Red River), Nanpan River (Pearl River) and Jinshajiang River (Yangtze River) and the plateau freshwater lakesrepresented by nine lakes (Luguhu Lake, Chenghai Lake, Erhai Lake, Dianchi Lake, Yangzonghai Lake, Fuxian Lake, Xingyun Lake, Qilu Lake, and Yilong Lake). The study of lignicolous freshwater fungi in Yunnan is conducive to clarifying the diversity, community composition, distribution pattern and influencing factors of freshwater fungi, and provides necessary support for the study of lignicolous freshwater fungi, including protection of the ecological environment and some feasibility suggestions to use lignicolous freshwater fungi in human welfare.

### Factors affecting the diversity and community of lignicolous freshwater fungi

There are many studies on lignicolous freshwater fungi from streams in tropical and temperate regions, involving species diversity and community differences, which indicate that taxa in temperate and tropical rivers rarely overlap (Hyde and Goh 1999; Cai et al. 2003c). Hyde et al. (2016a) reviewed the lignicolous freshwater fungi in Asia/Australia, attempted to study the distribution of fungi along the north–south latitude gradient. The effects of riparian vegetation, water pollution, sampling methods and global warming on the diversity of lignicolous freshwater fungi are discussed. The overall trend is that there are more lignicolous freshwater fungi in tropical streams, and there are more taxa per sample. The diversity of freshwater fungi and the community structure are influenced by many factors. Studies have pointed out that there is a positive correlation between the diversity of freshwater fungi and the diversity of riparian vegetation (Fabre 1996; Tsui et al. 2000; Laitung and Chauvet 2005; Lecerf et al. 2005; Vijaykrishna et al. 2006). However, other studies have not found the relationship between the diversity of riparian trees and the richness of fungal species (Wood-Eggenschwiler and Bärlocher 1983). Studies have also shown that afforestation does not have a consistent effect on freshwater fungal diversity (Bärlocher and Graça 2002; Ferreira et al. 2006). Water pollution affects the diversity and abundance of lignicolous freshwater fungi. For example, some industrial mining hazards reduce the abundance of freshwater fungal species and act as performance indicators (such as affecting the fungal spore yield and biomass and matrix decomposition efficiency) (Maltby and Booth 1991; Bermingham et al. 1996; Niyogi et al. 2002, 2009; Schlief et al. 2004; Lecerf and Chauvet 2008). Some studies have found that water eutrophication leads to a decline in the diversity of freshwater fungi (Lecerf and Chauvet 2008), but it does not seem that nutrient levels will directly inhibit certain species and lead to their extinction (Solé et al. 2008). Temperature is generally considered to be the most important environmental factor that affects metabolic functions and ultimately affects the growth and survival of microorganisms (Madigan et al. 2009). Global warming will lead to the reduction or extinction of freshwater fungi that grow in low-temperature environments. The direct main consequence of increase in the temperature is the faster decomposition of freshwater fungal substrates, resulting in a decrease in the supply of suitable substrates, and rapid deterioration of the ability of the substrates to maintain freshwater fungal colonies (Bärlocher et al. 2008). In addition, extreme weather events (such as record high or low temperatures, floods, and droughts) also affect the activities of aquatic fungi, which in turn will affect their distribution and occurrence, as well as the composition of the fungal community (Gerd-Joachim Krauss 2011).

### The potential value of lignicolous freshwater fungi

Lignicolous freshwater fungi are an important group in freshwater ecology. In addition to decomposing plant litter in rivers, they also have many potentials to offer. Fungi that grow in special environments are now producers of biologically active compounds. Their survival and the synthesis of new secondary metabolites and potential biologically active compounds that adapt to these conditions can be expected in these fungi with the greatest possibility (Grabley et al. 1999). Among the fungi from different environments, freshwater fungi have attracted renewed attention of the scientific community. The organic extracts of some freshwater fungi and isolated molecules show diverse biological activities against fungi and bacteria. The aromatic polyketide isolated from *Minutisphaera parafimbriatispora* (G156-4) showed moderate activity against *Staphylococcus aureus* and *Mycobacterium smegmatis* (Raja et al. 2015). Two new compounds isolated from *Xylomyces chlamydosporus* (H58-1) are active against *Fusarium verticillioides* (NRRL 25457) (Gloer et al. 2010). Two new metabolites isolated from *Delitschia corticola* (YMF 1.01111) are active against three fungal strains, *viz. Alternaria* sp., *Sclerotium* sp., and *Fusarium* sp., and three bacterial strains, *Bacillus cereus, B. laterosporus*, and *Staphylococcus aureus* (Sun et al. 2011). Some active substances are cytotoxic to cancer cells, such as the organic extracts of *Chaetomium* sp. (YMF 1.02105) from solid substrate fermentation are active against the growth of *Staphylococcus aureus* (ATCC 6538) and cancer cell lines A549 and MCF-7 (Shen et al. 2012). In addition, some bioactive substances are active against human pathogenic animals, for example, polyketide quinaphthin obtained from the fermentation broth of *Helicoon richonis* (SY034843), showed activity against two wall-less bacteria, *Acholeplasma laidlawii* (NCTC 10116) and *Mycoplasma gallisepticum* (NCTC 10115), and the human protozoan pathogen *Trichomonas vaginalis* (Fisher et al. 1988). The biologically active compounds produced by freshwater fungi have shown great potential from drug discovery point of view on countless different biological analyses.

Some lignicolous freshwater fungi can be used as biomarkers of changes in freshwater ecosystems under human pressure. They can segregate and detoxify heavy metals or other toxins and help to predict their resilience when challenged by selected human pressure (Gadd 2007). The generally expected response of species affected by climate is in gradience to higher altitudes and latitudes. If freshwater fungal species respond similarly, they will then provide a simple and inexpensive way to assess the progress of climate change and its impact on river communities (Krauss et al. 2011).

## Conclusion

At present, the lignicolous freshwater fungi that have been reported in Yunnan Province are mostly in *Dothideomycetes* (124 species) and *Sordariomycetes* (134 species), a few in *Eurotiomycetes* and *Leotiomycetes*, and rarely in *Orbiliomycetes* and *Pezizomycetes*. However, a large number of species in these fungal groups remain undiscovered. The freshwater fungi generally form small, fewer colonies and therefore are not easy to be found in the process of microscopic observations. In addition, some are difficult to cultivate on synthetic culture media. The study of lignicolous freshwater fungi in Yunnan has so far been mainly concentrated in northwestern Yunnan. Compared with lotic freshwater environment, there are relatively fewer studies on lentic waters such as plateau lakes. In addition, there are abundant plateau and alpine micro-water habitats in northwestern Yunnan (Liu et al. 2017), which contribute significantly to the formation of regional biodiversity patterns and thereby in the maintenance of regional ecological functions (Williams et al. 2004; Scheffer et al. 2006; Stokstad 2014; Liu et al. 2017). There is no credible research on lignicolous freshwater fungi related to these micro-water bodies.

High-throughput sequencing is almost a mature and rapid screening technology, which is widely used in various fields of biological research, including taxonomic identification. High-throughput sequencing provides a wide range of assistance in the study of fungi in various environments, such as oceans (Zhang et al. 2018; Xu et al. 1992; Yang et al. 2020), lakes (Antonelli et al. 2020; Guan et al. 2020), soil (Purahong et al. 2019; Zhang et al. 2019; Li et al. 2020b) and air (Aguayo et al. 2018; Woo et al. 2018). This can effectively solve the problem of the identification of unculturable lignicolous freshwater fungi.
